# Environmental neurotoxin dieldrin induces apoptosis via caspase-3-dependent proteolytic activation of protein kinase C delta (PKCdelta): Implications for neurodegeneration in Parkinson's disease

**DOI:** 10.1186/1756-6606-1-12

**Published:** 2008-10-22

**Authors:** Anumantha G Kanthasamy, Masashi Kitazawa, Yongjie Yang, Vellareddy Anantharam, Arthi Kanthasamy

**Affiliations:** 1 Parkinson's Disorder Research Laboratory, Iowa Center for Advanced Neurotoxicology, Department of Biomedical Sciences, Iowa State University, Ames, IA, USA

## Abstract

**Background:**

In previous work, we investigated dieldrin cytotoxicity and signaling cell death mechanisms in dopaminergic PC12 cells. Dieldrin has been reported to be one of the environmental factors correlated with Parkinson's disease and may selectively destroy dopaminergic neurons.

**Methods:**

Here we further investigated dieldrin toxicity in a dopaminergic neuronal cell model of Parkinson's disease, namely N27 cells, using biochemical, immunochemical, and flow cytometric analyses.

**Results:**

In this study, dieldrin-treated N27 cells underwent a rapid and significant increase in reactive oxygen species followed by cytochrome c release into cytosol. The cytosolic cytochrome c activated caspase-dependent apoptotic pathway and the increased caspase-3 activity was observed following a 3 hr dieldrin exposure in a dose-dependent manner. Furthermore, dieldrin caused the caspase-dependent proteolytic cleavage of protein kinase C delta (PKCδ) into 41 kDa catalytic and 38 kDa regulatory subunits in N27 cells as well as in brain slices. PKCδ plays a critical role in executing the apoptotic process in dieldrin-treated dopaminergic neuronal cells because pretreatment with the PKCδ inhibitor rottlerin, or transfection and over-expression of catalytically inactive PKCδ^K376R^, significantly attenuates dieldrin-induced DNA fragmentation and chromatin condensation.

**Conclusion:**

Together, we conclude that caspase-3-dependent proteolytic activation of PKCδ is a critical event in dieldrin-induced apoptotic cell death in dopaminergic neuronal cells.

## Background

Epidemiological studies of Parkinson's disease (PD) over the past decade have promoted the conclusion that idiopathic, geriatric-onset PD is an environmentally-mediated neurodegenerative disorder [1-5]. PD-associated factors most often cited include residence in a rural area, use of well water as a drinking water source, and occupational use of pesticides, all of which are linked to pesticide exposures. These have been reported in numerous epidemiological studies [6-17]. A landmark epidemiology study by Tanner and colleagues [18] of nearly 20,000 twin pairs from a WWII veterans health care database determined that no clear genetic correlate exists to explain the incidence of PD and concluded that PD is an environmentally-mediated disorder. Postmortem studies of PD patients have reported significantly higher brain concentrations of chlorinated hydrocarbons, particularly cyclodiene insecticides [19-21], further suggesting a direct link between environmental exposure to neurotoxicants and PD.

Cyclodiene insecticides are heavily chlorinated toxicants that act primarily as antagonists of the GABA_A _receptor ionophore [22,23]. Since the majority of GABA projections in the brain are inhibitory in function, cyclodienes are pharmacologically defined as pro-convulsant chemicals [23-25]. Pharmacokinetically, cyclodienes and similar lipophilic chlorinated cage toxicants, collectively termed polychlorocycloalkanes, accumulate in fatty tissues and the brain [26,27]. Dieldrin, specifically, is one of the most environmentally persistent insecticides known [28]. Polychlorocycloalkanes were used extensively due to their excellent latent-kill activity against crop and structural pests and their low cost. However, bioaccumulation and biomagnification in non-target species led to the ban of these chemicals in the 1970s, with a few exceptions (e.g., g-HCH, endosulfan, methoxychlor). Approximately 3 billion tons of these chemicals have been manufactured and used commercially for insect control to date [29].

Despite the current restricted use of polychlorocycloalkanes in western countries, humans continue to be exposed through either direct contact with environmental residues, exposure to contaminated ground water, or consumption of imported products from countries where these chemicals are still legal for agricultural and industrial use. Daily dietary exposure to dieldrin, according to a study of 120,000 U.S. adults, is estimated to be in excess of EPA minimum safety standards [30].

Attempts to link dieldrin to emerging models of disease progression in idiopathic PD have been reported by our laboratory [31,32] and others [33-35]. Previously, we demonstrated the existence and regulation of a selective toxicant-evoked apoptotic pathway in PC12 cells which incorporates a signal amplification loop between caspase-3 and PKCδ [32]. Herein, we report further characterization of dieldrin-specific pro-apoptotic effects in a rat mesencephalic cell line (N27) with dopaminergic characteristics. The present work strongly supports results reported in PC12 cell studies that indicated dieldrin initiates apoptosis in cells by a mitochondrial mechanism that facilitates early onset reactive oxygen species generation, cytochrome c release to the cytoplasm, caspase cascade activation, and PKCδ cleavage and activation. The central role of PKCδ linking initiation and end-point effects in dieldrin-induced apoptosis is supported by evidence presented and discussed in the present work.

## Materials and methods

### Materials

Dieldrin, Hoechst 33342, and mouse monoclonal anti-β-actin antibody were purchased from Sigma (St. Louis, MO, USA). Caspase-3 substrate, Ac-DEVD-AMC, was purchased from Bachem Biosciences, Inc. (King of Prussia, PA). Caspase-3 specific inhibitor, Z-DEVD-FMK, was purchased from Alexis Biochemicals (San Diego, CA). Hydroethidine was purchased from Molecular Probes (Eugene, OR, USA). Rabbit polyclonal anti-PKCδ antibody was purchased from Santa Cruz Biotechnology, Inc. (Santa Cruz, CA). ECL Western blotting analysis kit was purchased from Amersham Pharmacia Biotech, Inc. (Piscataway, NJ). Cell Death Detection Elisa Plus Assay kit was purchased from Roche Molecular Biochemicals (Indianapolis, IN). Cytochrome c ELISA kit was obtained from MBL International Corp. (Watertown, MA). All tissue culture supplies were purchased from Gibco-BRL (Gaithersburg, MD, USA). Other routine laboratory reagents were purchased from Fisher Scientific (Pittsburg, PA, USA). Plasmids, PKCδ^K376R^-GFP fusion protein, and pEGFP-N1 were kind gifts from Dr. Stuart H. Yuspa, National Cancer Institute (Bethesda, MD). The immortalized rat mesencephalic (N27) cell line was a kind gift from Dr. Kedar N. Prasad, University of Colorado Health Sciences Center (Denver, CO).

### Animals

Adult male Sprague Dawley rats (125–150 g; Zivic Miller Laboratory, Alison Park, PA) were used in all experiments with animal tissues. Rats were housed one per cage in a temperature-controlled room (23°C) with a 12:12 L:D cycle. Animals were fed standard laboratory diet and water *ad libitum*. Experimental procedures used here were approved by the Institutional Animal Care and Use Committee at Iowa State University. The Iowa State University vivarium is an AAALAC approved facility.

### Stable transfection

Plasmid pPKCδ^K376R^-GFP encodes protein kinase Cδ-GFP fusion protein; K376R refers to the mutation of the lysine residue at position 376 to arginine in the catalytic site of PKCδ, rendering it inactive [36]. Plasmid pEGFP-NI encodes the green fluorescent protein alone and was used as vector control. N27 cells stably expressing pEGFP-NI and pPKCδ^K376R ^were established using Lipofectamine Plus reagent, as per the procedure recommended by the manufacturer and described previously (51). Stable cell lines were maintained in medium containing 200 μg/ml hygromycin.

### Cell lines

Immortalized rat mesencephalic cells (1RB_3_AN_27_, abbreviated here as N27 cells) were grown in RPMI medium supplemented with 10% fetal bovine serum, 1% L-glutamine, penicillin (100 U/ml), and streptomycin (100 U/ml), and maintained at 37°C in a humidified atmosphere of 5% CO_2 _[37,38]. Vector-transfected (N27-GFP) and PKCδ dominant negative mutant (N27-PKCδ^K376R^) cells were maintained in serum containing growth medium with 200 μg/ml hygromycin.

### Isolation of cytosolic fraction in N27 cell homogenates

Cytosolic fractions were isolated from untreated and dieldrin-treated N27 cells as per previously published procedures (51). Briefly, cells were pelleted by centrifugation at 200 × g for 10 min at 4°C. The cell pellet was washed once with ice-cold PBS and resuspended in 2 ml homogenization buffer (20 mM Tris HCl, 2 mM EDTA, 10 mM EGTA, 2 mM dithiothreitol, 1 mM phenylmethylsulfonyl fluoride, 25 μg/ml aprotonin, and 10 μg/ml leupeptin). The suspension was then sonicated for 10 sec and centrifuged at 100,000 × g for 1 hr at 4°C. Resulting supernatant was used as a cytosolic fraction. The protein concentration of each cytosolic fraction was determined using a Bradford protein assay dye reagent (BioRad Laboratories; Hercules, CA, USA). Cytosolic fractions were stored frozen at -80°C and used subsequently for biochemical and Western blot experiments. Samples were diluted with homogenization buffer according to the protein concentration estimated by the assay to equalize protein concentrations for gel loading. Each sample was then mixed with 2× gel loading buffer containing 10% SDS and 200 μM DTT and placed in boiling water for 5 min.

### Treatment paradigm

After 2–4 days in culture, N27 cells were harvested and resuspended in serum-free growth medium at a cell density of 1–3 × 10^6^/ml. Cell suspensions were treated with DMSO (0.1% final concentration) or dieldrin (30–300 μM) over a period of 5 min to 3 hr at 37°C. In inhibitor studies, Z-DEVD-FMK (caspase-3-specific inhibitor, 50 μM), was added 30 min prior to the addition of dieldrin. The reaction samples were removed at various time points, centrifuged at 200 × g (5 min, 4°C), and cell pellets were used for assessing cytochrome c release, caspase-3 enzymatic activities, PKCδ cleavage, and DNA fragmentation. Cell samples used for flow cytometry were further treated with visualization flourometric chemicals, as described in the methods below.

### Brain slice preparation and treatment

Sprague Dawley male rats (125–150 g) were euthanized by ether and decapitated. Brains were removed by brain case dissection to a cold table, dura and pia mater were removed by forceps, and brains were rinsed with 0.9% sterile saline. Brain sections (300 μm) were cut in 4°C carboxygenerated (5% CO_2_/95% O_2_) artificial cerebrospinal fluid slicing medium (1.4 mM KCl, 685 μM NaH_2_PO_4_, 14 mM NaHCO_3_, 2 mM CaCl_2_, 1.2 mM MgSO_4_, 50 mM sucrose, and 2.5 mM dextrose) using a Lancer Vibratome (model 1000; The Vibratome Co., St. Louis, MO, USA). Sections were transferred to carboxygenerated artificial cerebrospinal fluid (ACSF; 126 mM NaCl, 2.5 mM KCl, 1.25 mM NaH_2_PO_4_, 26 mM NaHCO_3_, 2 mM CaCl_2_, 1.2 mM MgSO_4_, and 2.5 mM dextrose) and allowed to recover from trauma for 2 hr at 37°C prior to treatment with toxicants. At 2 hr, the incubation medium was refreshed with 37°C carboxygenerated ACSF and DMSO (0.033% final concentration), or DMSO containing dieldrin (30–100 μM final concentration) was added to the medium and incubated with slices for 3 hr at 37°C. Following incubation, slices were removed to 1.5 ml tubes, centrifuged briefly at 1000 × g. The supernatant was discarded and tissues were prepared for Western blot by Dounce homogenization (15 strokes) in a modified lysis buffer (25 mM HEPES, 100 μM Na_2_VO_4_, 300 μM NaCl, 1.5 mM MgCl_2_, 200 μM EDTA, 50 mM dithiothreitol, 10 μl Triton X-100, 20 mM NaF, 20 mM β-glycerophosphate, 1 mM phenylmethylsulfonyl fluoride, 25 μg/ml aprotinin, and 50 μg/ml leupeptin) at 400 μl/slice. Homogenates were centrifuged (12,000 × g, 20 min, 4°C) and protein concentrations of supernatants were determined using a Bradford protein assay dye reagent (BioRad Laboratories, Hercules, CA, USA). Cytosolic fractions were stored.

### Reactive oxygen species (ROS) flow cytometry

Flow cytometry analysis was performed on a Becton Dickenson FACScan™ flow cytometer (Becton Dickenson, San Francisco, CA), as described previously (51). Hydroethidine, a sodium borohydride-reduced derivative of ethidium bromide, was used to detect ROS production, specifically O_2_^-^inside the cell [39]. Hydroethidine loaded to cells binds to cellular macromolecules and reacts with O_2_^- ^as it is generated, converting hydroethidine to ethidium bromide, increasing red fluorescence (620 nm). A 15-mW air-cooled argon-ion laser was used as an excitation source for hydroethidine at 488 nm, and the optical filter was 585/42 nm bandpass. Cells were detected and distinguished from the background by forward-angle light scattering (FSC) and orthogonal light scattering (SSC) characteristics. All the flow cytometric data were analyzed by Cellquest™ data analysis software to determine significant increases or decreases in fluorescence intensity.

### Cytochrome c release assay

Dieldrin-induced cytochrome c release was measured using a cytochrome c ELISA kit, as described previously [37]. Briefly, N27 cells (5 × 10^6 ^cells) were resuspended in serum-free RPMI-1640. Cell suspensions were exposed to 100 μM or 300 μM dieldrin for 15–30 min at 37°C. After exposure, cells were collected, washed once with ice-cold phosphate-buffered saline (PBS; pH 7.4), and resuspended in 1 ml of ice-cold homogenization buffer (10 mM Tris HCl pH 7.5, 0.3 M sucrose, 1 mM phenylmethylsulfonyl fluoride, 25 μg/ml aprotinin, 10 μg/ml leupeptin). Following homogenization, cells were centrifuged at 10,000 × g for 60 min at 4°C. Resulting supernatants were collected as cytoplasmic fractions and used to measure cytochrome c release by the cytochrome c ELISA assay kit, strictly following the protocol provided by the manufacturer (MBL, Watertown, MA, USA). Optical density of each well was then measured at 450 nm using a microplate reader (Molecular Devices Corp., Sunnyvale, CA, USA). The cytochrome c concentration was calibrated from a standard curve based on reference standards.

### Caspase-3 activity

Caspase activities were determined as previously described [37]. Briefly, after exposure to dieldrin, cells were washed once with PBS and resuspended in lysis buffer containing 50 mM Tris/HCl (pH 7.4), 1 mM EDTA, 10 mM EGTA, and 10 μM digitonin. Cells were then incubated at 37°C for 20–30 min to allow complete lysis. Lysates were quickly centrifuged and cell-free supernatants were incubated with 50 μM Ac-DEVD-AMC (caspase-3 substrate) at 37°C for 1 hr. Caspase activity was then measured using a microplate reader (Molecular Devices Corp., Sunnyvale, CA) with excitation at 380 nm and emission at 460 nm. Caspase activity was expressed as fluorescence unit (FU) per mg protein per hr.

### Western blot

Samples were diluted to protein concentrations appropriate for gel loading and boiled for 5 min in 2× gel loading buffer containing 10% SDS and 20 mM dithiothreitol. Samples were stored at -80°C until used for Western blot analysis. Cytoplasmic fractions or brain tissue samples containing equal amounts of protein (5–10 μg) were loaded in each lane and separated on a 10% SDS-polyacrylamide gel. Proteins were then transferred to nitrocellulose membranes by electro-blotting overnight (4°C, 25 V). Non-specific binding sites were blocked by treating the nitrocellulose membranes with 5% non-fat dry milk powder for 2 hr prior to treatment with primary antibodies. Nitrocellulose membranes containing the proteins were incubated with rabbit anti-PKCδ for 1 hr at RT (1:2000 dilution). Primary antibody treatments were followed by treatment with secondary HRP-conjugated anti-rabbit IgG (1:2000 dilution) for 1 hr at RT. Secondary antibody-bound proteins were detected using Amersham's ECL chemiluminescence kit. To confirm equal protein loading, blots were re-probed with a β-actin antibody (1:5000 dilution). Gel photographs were taken with a gel imaging system and quantification of bands was performed using Scion Image.

### Annexin V and propidium iodide flow cytometry

Flow cytometry analysis of apoptotic and necrotic N27 cells following a 3 hr exposure to dieldrin (100 μM) was performed by Annexin V-FTIC and propidium iodide (PI) staining kit (BD PharMingen), as per the manufacturer's specifications and as described previously [51]. Annexin V binds to phosphatidylserine (PS) and other negatively charged phospholipids, producing fluorescence primarily indicative of PS translocation from the inner to the outer cell membrane leaflet, reflective of aminophospholipid translocase activity in apoptotic cells [40]. PI is a nucleic acid dye that penetrates the nuclear envelope of necrotic cells and was used here as a counter stain to differentiate between live, apoptotic, late stage apoptotic/early stage necrotic, and necrotic cells. Flow cytometry analysis was performed on a Becton Dickenson FACScan™ flow cytometer (Becton Dickinson, San Francisco, CA). N27 cells were washed twice with cold phosphate-buffered saline (pH 7.4) and resuspended in a binding buffer (10 mM HEPES, 140 mM NaCl, 2.5 mM CaCl_2_; pH 7.4) at a concentration of 0.5 × 10^6 ^cells/ml. Cell aliquots of 100 μl were incubated with Annexin V-FITC (5 μl) and PI (2 μl) for 15 min at RT in the dark. After 15 min, incubates were diluted with 400 μl of binding buffer and analyzed by flow cytometry. A 15-mW air-cooled argon-ion laser was used as an excitation source for Annexin V-FITC at 488 nm with optical filter at 530/15 nm bandpass. PI fluorescence was measured with the optical filter at 650/42 nm bandpass. Cells were detected and distinguished from the background by forward-angle light scattering (FSC) and orthogonal light scattering (SSC) characteristics. All the flow cytometric data were analyzed by Cellquest™ data analysis software to determine the significant increases or decreases of fluorescence intensity.

### DNA fragmentation analysis

DNA fragmentation assay was performed using a Cell Death Detection Elisa Plus Assay kit (51). N27 cells were exposed to DMSO (0.1% final concentration) or DMSO containing dieldrin (30–100 μM) for 3 hr at 37°C. Following treatment, cells were centrifuged at 200 × g for 5 min at 4°C and washed once with 1× phosphate-buffered saline (pH 7.4). Cells were then incubated with a lysis buffer (supplied with the kit) at RT for 30 min. Incubates were centrifuged at 10,000 × g for 20 min at 4°C and 20 μl aliquots of supernatant were dispensed to streptavidin-coated 96 well microtiter plates followed by addition of 80 μl of antibody cocktail. Plates were incubated for 2 hr at RT with mild shaking. The antibody cocktail consisted of a mixture of anti-histone biotin and anti-DNA-HRP directed against various histones and antibodies to both single strand DNA and double strand DNA, which are major constituents of the nucleosomes. After incubation, unbound components were removed by washing with the incubation buffer supplied with the kit. Quantitative determination of the amount of nucleosomes retained by anti-DNA-HRP in the immunocomplex was determined spectrophotometrically with 2,2'-azino-di-(3-ethylbenzthiazoline sulfonate (6)) diammonium salt (ABTS) as an HRP substrate (supplied with the kit). Measurements were made at 405 nm against an ABTS solution as a blank (reference wavelength ~490 nm) using a Molecular Devices Spectramax Microplate Reader. The data were analysed using Graphpad Prism 4.0 software and expressed as percentage of DNA fragmentation observed in vehicle (0.1% DMSO)-treated cells.

### Immunocytochemistry

N27 cells were grown on collagen (6 μg/cm^2^) coated slides for 2–3 days in a 37°C, 5% CO_2 _incubator. Cells were washed twice with phosphate-buffered saline (pH 7.4) and treated for 3 hr with DMSO (0.1% final concentration) or DMSO containing dieldrin (100 μM). Cells were again washed with phosphate-buffered saline and fixed with 10% buffered formaldehyde for 30 min at room temperature, followed by staining with Hoechst 33342 (10 μg/ml) for 3 min in the dark. Cells stained with Hoechst 33342 dye fluoresce bright blue upon binding to DNA in the nucleus. The nucleus of apoptotic cells exhibits strong blue staining in a heterogeneous and patchy pattern, indicative of chromatin condensation, whereas the nucleus of non-apoptotic cells exhibits a more diffused, weak and homogenous staining [41,42]. Slide-mounted cells were observed under a Nikon DiaPhot microscope under UV illumination, and pictures were captured with a SPOT digital camera (Diagnostic Instruments, Sterling Heights, MI).

### Data analysis

Data were first analyzed using one-way ANOVA. Dunnett's post-test or Bonferroni's multiple comparison test was then performed to compare treated samples, and p < 0.05 was considered significant.

## Results

### Dieldrin-induced reactive oxygen species (ROS)

**Figure 1:**
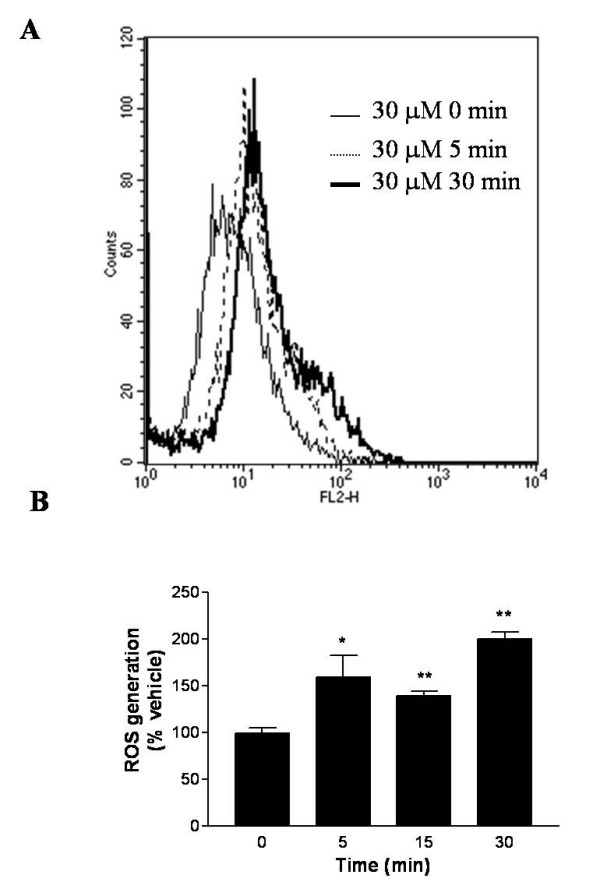
**Dieldrin-induced ROS generation in N27 cells**. N27 cells (~1 × 10^6 ^cells/ml) were treated with 30 μM dieldrin for 0–30 min. Hydroethidine fluorescence intensity was measured at various time points by flow cytometry. Representative shift of fluorescent intensity during dieldrin treatment (A). Quantitative analysis of ROS generation deduced from Fig. 1A (B). Data represent the mean ± SEM for three separate experiments performed in triplicate. Significance was determined by ANOVA followed by Dunnett's post-test between the vehicle-treated group and dieldrin-treated group (*p < 0.05 and **p < 0.01).

Exposure of N27 cells to dieldrin resulted in a rapid, transient increase in generation of ROS as measured by flow cytometric analysis of hydroethidium dye oxidation (Fig. 1A). A significant shift of fluorescent intensity indicates the massive generation of intracellular ROS in a time-dependent manner. Moderate concentrations of dieldrin (30 μM) produced a significant time-dependent increase of approximately 65% in ROS levels 5 min after treatment (p < 0.01), which appeared to reach signal saturation by 15 min, at approximately 200% of vehicle control (Fig. 1B). Comparison of exposure of N27 cells to various concentrations of dieldrin (30–200 μM) indicated that similar levels of ROS generation were reached at 5 min post-treatment (~145–165% vehicle control) and subsequently reversed at concentrations of dieldrin greater than 100 μM beyond 5 min exposure (data not shown).

### Dieldrin promotes mitochondrial cytochrome c release

**Figure 2:**
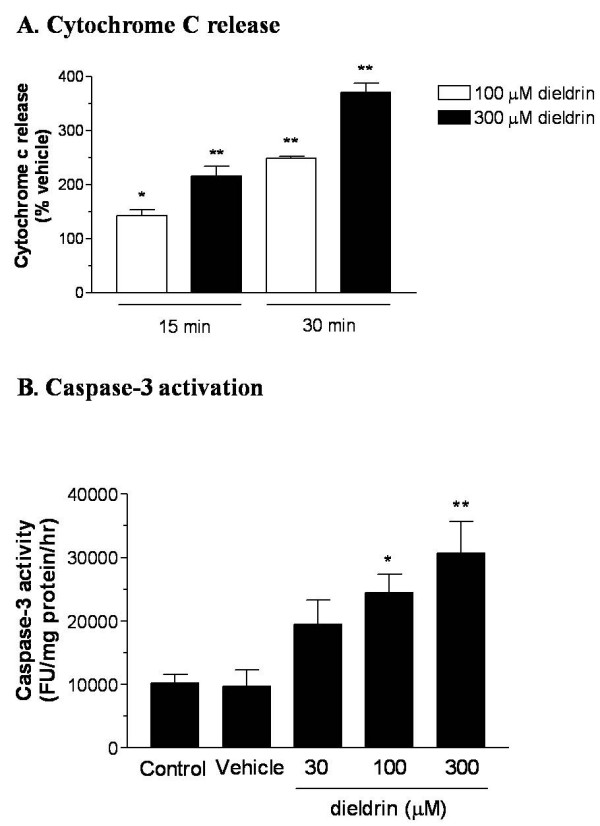
**Dieldrin-induced increases in cytosolic cytochrome c and caspase-3 activity in N27 cells**. In A, N27 cells (5 × 10^6 ^cells) were exposed to 100 or 300 μM dieldrin for 15–30 min. The mitochondria-free cytosolic fraction was collected as described in "Materials and Methods," and cytosolic cytochrome c was measured using ELISA cytochrome c assay. Data represent the mean ± SEM for three separate experiments performed in triplicate. Significance was determined at *p < 0.05 or **p < 0.01 compared with the vehicle-treated group at each time point. In B, N27 cells (2 × 10^6 ^cells/ml) were exposed to 30–300 μM dieldrin for 3 hr at 37°C, and caspase-3 activity was measured using the caspase-3 specific substrate Ac-DEVD-AMC, as described in the "Materials and Methods." The data are expressed as fluorescent unit (FU) per mg protein per hr of incubation. Each point represents mean ± SEM from two separate experiments in triplicate. Significance was determined at *p < 0.05 or **p < 0.01 compared with vehicle-treated cells.

Dieldrin-mediated cytochrome c release, an early event in apoptosis, was determined by ELISA assay. As shown in Fig. 2A, dieldrin treatment induced time- and dose-dependent increases in the appearance of cytochrome c in the cytosol over a 30 min exposure period. Dieldrin significantly increased cytochrome c release by 50% and 140% at 15 and 30 min post-treatment, respectively. Exposure of N27 cells to 300 μM dieldrin evoked significant increases in accumulation of cytosolic cytochrome c by 110% and 260% following 15 min and 30 min of dieldrin exposure, respectively.

### Dieldrin-mediated activation of caspase-3

Dieldrin increased caspase-3 enzyme activity, an important effector caspase in apoptosis, in a dose-dependent manner after 3 hr of exposure, as measured by Ac-DEVD-AMC fluorometry (Fig. 2B). Caspase-3 activity increased by 210%, 260%, and 340% of vehicle control following treatment with 30, 100, and 300 μM dieldrin, respectively. No significant vehicle effect (DMSO, 0.2% final concentration) was observed, suggesting that measured effects were directly attributable to dieldrin exposure. Concentrations of dieldrin above 100 μM appeared to produce similar levels of caspase-3 activity, whereas 30 μM dieldrin-induced increases in caspase-3 activity were lower but greater than the vehicle control level.

### Caspase-3-dependent dieldrin-induced cleavage and activation of PKCδ

**Figure 3:**
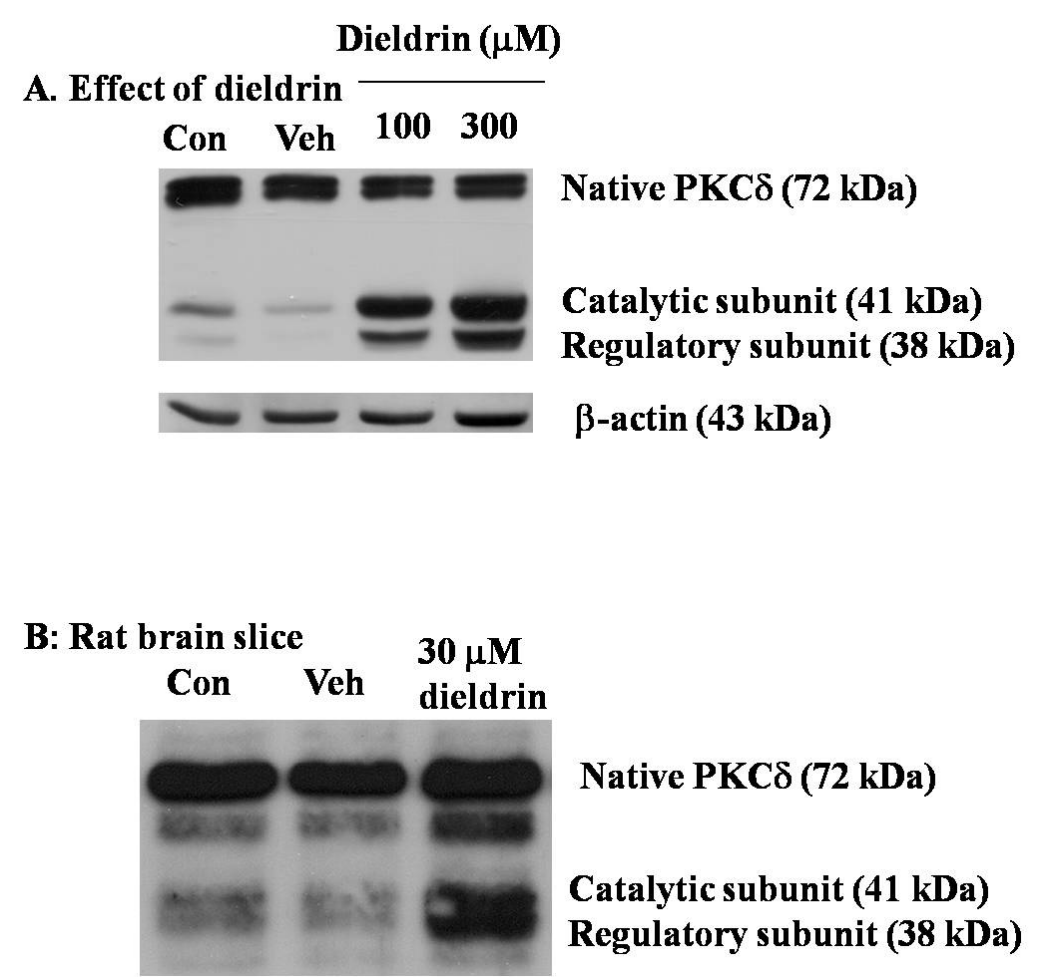
**Proteolytic cleavage of PKCδ following dieldrin treatment in N27 cells**. In A, N27 cells (~1 × 10^7 ^cells) were exposed to 100 μM or 300 μM dieldrin for 3 hr at 37°C, and cytosolic proteins were collected as described in "Materials and Methods." Approximately 5 μg of cytosolic proteins were resolved on 10% SDS-polyacrylamide gel, revealing native PKCδ (72 kDa), the catalytic subunit (41 kDa) and the regulatory subunit (38 kDa) of proteolytically cleaved PKCδ. In B, rat brain slices were exposed to 30 μM dieldrin for 3 hr. PKCδ was detected by Western blot and equal protein loading was confirmed by reprobing with β-actin (43 kDa). Veh represents 0.2% DMSO.

**Figure 4:**
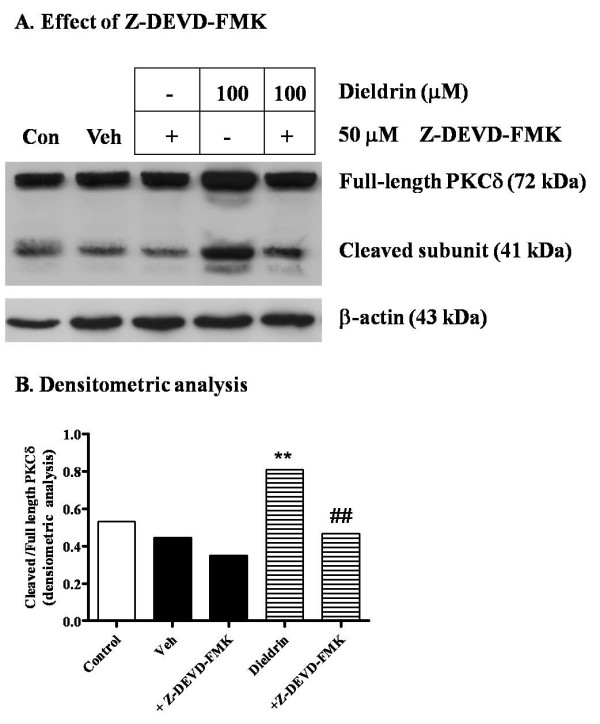
**Effect of caspase-3 inhibitor on dieldrin induced PKCδ proteolytic cleavage**. In A, N27 cells were pretreated with caspase-3 specific inhibitor, Z-DEVD-FMK (50 μM), for 30 min, and then exposed to 100 μM dieldrin for another 3 hr and cytosolic proteins were collected as described in "Materials and Methods." PKCδ was detected by Western blot, and equal protein loading was confirmed by reprobing with β-actin (43 kDa). Veh represents 0.2% DMSO. In B, Densitometric data of cleaved (41 kDa) and full-length (72 kDa) PKCδ bands in A. Densitometric analysis was performed to determine the level of inhibition of proteolytic cleavage of PKCδ by caspase-3 inhibitor. Density of each band was normalized to 43 kDa β-actin band prior to analysis. The data are expressed as a ratio of cleaved versus full-length. **p < 0.01 compared with vehicle-treated group and ##p < 0.01 compared with dieldrin-treated group.

Dieldrin at 100 and 300 μM induced maximum increases in caspase-3 activity by increasing the cleavage and activation of PKCδ in a concentration-dependent manner 3 hr following treatment, as measured by Western blot analysis (Fig. 3A). Previously, we and others have shown that PKCδ is selectively activated by caspase-3 under conditions of toxicant exposure [37,43,44]. Pretreatment with a selective caspase-3 inhibitor, Z-DEVD-FMK (50 μM), for 30 min prior to 3 hr treatment of N27 cells with 100 μM dieldrin markedly reduced PKCδ cleavage and activation (Fig. 4A), approximating basal levels of cleaved products observed in controls. As shown in Fig. 4B densitometric analysis of 41 kDa cleaved and 72 kDa full-length PKCδ bands in Fig 4A revealed an 80% increase in cleaved PKCδ band in dieldrin-treated samples compared to vehicle-treated samples. Whereas pretreatment with Z-DEVD-FMK almost completely prevented dieldrin induced increase in cleaved PKCδ levels. The densitometric data where normalized to 43 kDa β-actin band prior to analysis of cleaved and full-length PKCδ bands.

### Dieldrin promotes PKCδ cleavage in brain tissue

Preliminary results from incubations of 300 μm coronal sections of rat midbrain tissue with concentrations of dieldrin were similar to results in N27 cells. Dieldrin at 30 μM produced a 75.3% increase in cleaved products of PKCδ (Fig. 3B), reminiscent of changes observed with 100 μM dieldrin in N27 cells (see Fig. 3A), and suggested that similar apoptotic cell death processes may be activated *in situ *following dieldrin exposure.

### Dieldrin induces apoptotic cell death in N27 cells

**Figure 5:**
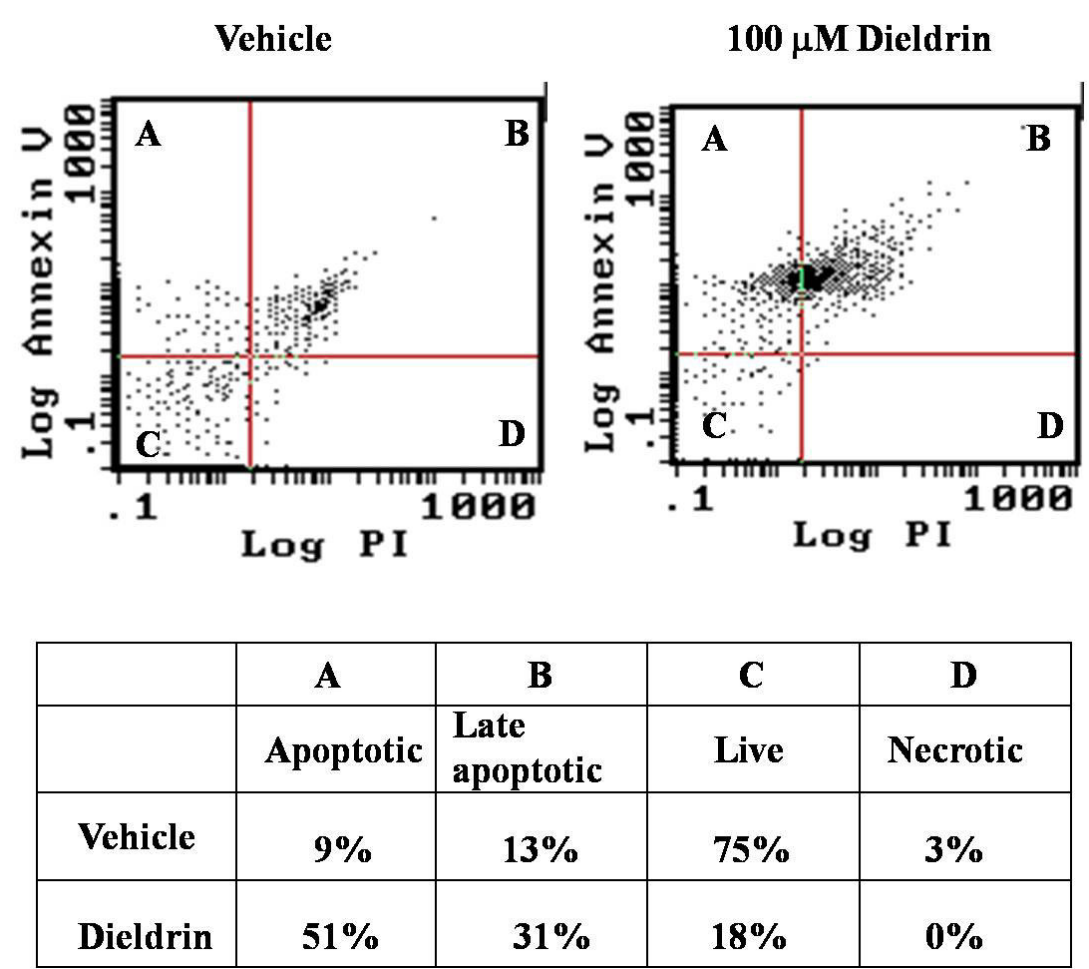
**Dieldrin-induced apoptosis in N27 cells**. N27 cells (~1 × 10^6 ^cells/ml) were treated with 100 μM dieldrin for 3 hr, and apoptotic cells were detected by flow cytometry as described in "Materials and Methods." Dual staining of cells with annexin-V-FITC and propidium iodide enabled categorization of cells into four regions (A, B, C, and D). Region A was apoptotic cells, B was apoptotic and necrotic cells, C was live or healthy cells, and D was necrotic cells. The experiment was repeated three times and data represent the average of each region.

Flow cytometric analysis of N27 cells incubated 3 hr with DMSO (0.1% final concentration) or DMSO containing dieldrin (100 μM) revealed marked increases in both apoptotic (annexin V, 51%) and apoptotic/necrotic or late apoptotic (annexin V and propidium iodide, 31%) indices, whereas vehicle-treated cells did not have an increased apoptotic level (9% and 13% apoptotic and late apoptotic, respectively) (Fig. 5). Chi square analysis of the distribution of annexin V FITC and propidium iodide positive cells indicated a positive trend toward apoptosis in dieldrin-treated N27 cells (χ^2 ^= 69.12, p < 0.0001).

### PKCδ mediates dieldrin-induced DNA fragmentation in N27 cells

**Figure 6:**
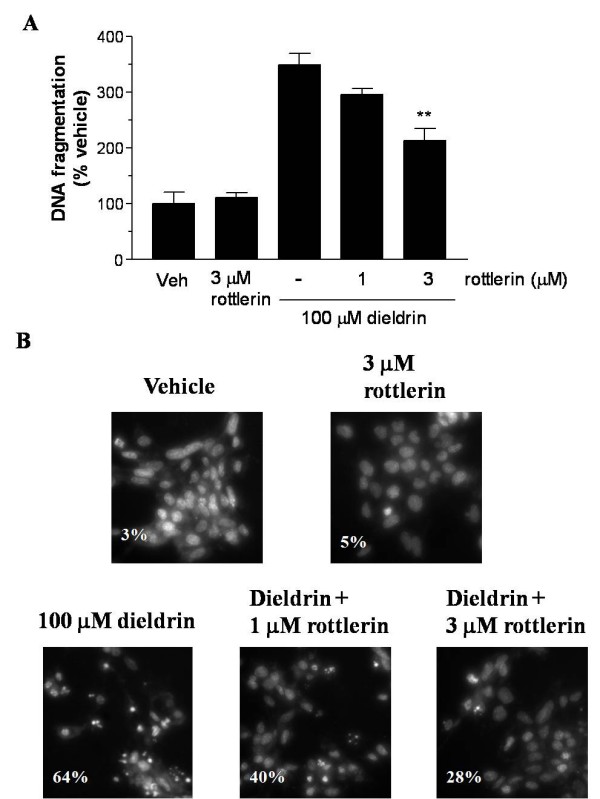
**PKCδ mediates dieldrin-induced DNA fragmentation and nuclear condensation in N27 cells**. N27 cells were pretreated with 1–3 μM rottlerin for 30 min, and then treated with 100 μM dieldrin for another 3 hr. In A, DNA fragmentation was quantitatively measured by ELISA DNA fragmentation assay kit. Each bar represents mean ± SEM for two separate experiments in triplicate. **p < 0.01 compared with dieldrin-treated group. In B, chromatin condensation was observed using Hoechst 33342 staining. The percentage of nuclear condensation was calculated by counting positive cells in three to five randomly selected regions. The experiment was repeated three times, and similar results were obtained.

To further confirm the results obtained by flow cytometry and to assess the involvement of PKCδ. in mediation of apoptosis, a quantitative DNA fragmentation assay was performed. N27 cells were treated with 100 μM dieldrin for 3 hr. N27 cells were also pretreated with 1–3 μM rottlerin for 30 min prior to dieldrin exposure As shown in Fig. 6A, dieldrin treatment alone induced > 3-fold increase in DNA fragmentation, and rottlerin dose-dependently blocked dieldrin-induced increases in DNA fragmentation. We also confirmed DNA fragmentation by qualitative analysis of apoptosis. Hoechst 33342 staining showed nuclear condensation, one of the distinct morphological changes during apoptosis, following 3 hr dieldrin exposure (Fig. 6B). Pretreatment with rottlerin remarkably reduced dieldrin-induced chromatin condensation. As shown in Fig. 6B, rottlerin dose-dependently protected against dieldrin-induced chromatin condensation. The levels of chromatin condensation observed by Hoechst 33342 were 64%, 40%, and 28% in 100 μM dieldrin only, dieldrin + 1 μM rottlerin, and dieldrin + 3 μM rottlerin, respectively. Together, these results suggest that proteolytic activation of PKCδ plays an important role in execution of apoptosis.

### Catalytically inactive PKCδ^K376R ^mutant rescues N27 cells from dieldrin-induced DNA fragmentation

**Figure 7:**
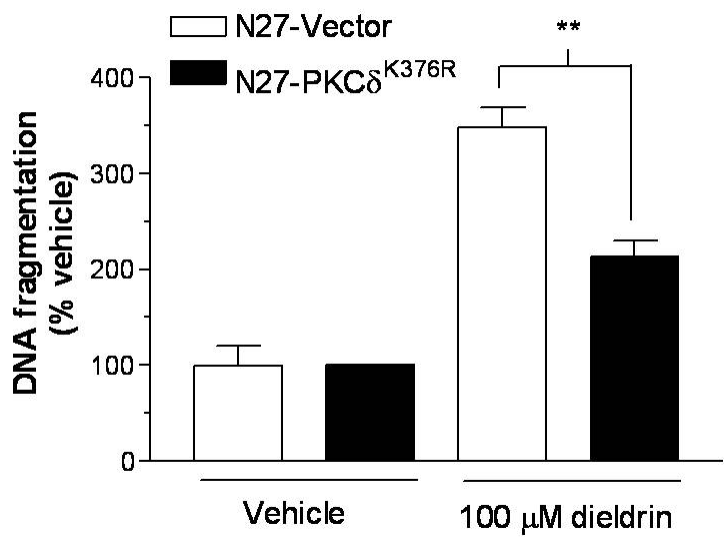
**The role of PKCδ in dieldrin-induced DNA fragmentation and nuclear condensation in N27 cells**. GFP-expressing vector cells and PKCδ^K376R^-transfected N27 cells were exposed to 100 μM dieldrin for 3 hr. DNA was extracted and DNA fragmentation was measured using ELISA DNA fragmentation assay kit, as described in "Materials and Methods." Each bar represents mean ± SEM. Significance was determined at **p < 0.01 compared with vehicle-treated cells or between indicated groups.

Measurement of DNA fragmentation by the ELISA method in N27-GFP and N27-PKCδ^K376R ^cells following 3 hr dieldrin exposure indicated a significant increase in DNA fragmentation (Fig. 7). Maximal increases (350%) in DNA fragmentation were observed with N27^GFP ^exposed to 100 μM dieldrin compared to only a 212% increase in DNA fragmentation observed in N27-PKCδ^K376R ^cells. These results suggest that N27-PKCδ^K376R ^cells were resistant to dieldrin-induced DNA fragmentation

## Discussion

Results presented here further support our hypothesis that dieldrin contributes to apoptotic cell death in dopaminergic neuronal cells. We and others previously demonstrated the selective toxicity of dieldrin on dopaminergic cells [31,45] as well as characterized the subsequent signaling cell death mechanism in dopaminergic PC12 cells [32]. Here we demonstrated that the cell death pathway observed in dopaminergic neuronal cells following acute exposure to dieldrin was identical to that observed in PC12 cells, i.e., i) initial and rapid increase in reactive oxygen species (ROS), ii) possible mitochondrial damage and subsequent release of cytochrome c, iii) caspase-3 activation and proteolytic cleavage of PKCδ, and iv) apoptotic cell death as a result of activation of these pro-apoptotic molecules.

Generation of ROS was a rapid response to dieldrin toxicity. Within 5 min after the exposure, cells increased intracellular ROS by 50% from the basal level, suggesting dieldrin somehow interacts with certain cellular molecules (possibly mitochondrial proteins responsible for cellular respiration) to potentiate the production of ROS as soon as it gets into the cells. Dieldrin reportedly inhibits the mitochondrial electron transport system (ETS) near the complex III [46]. Termination of ETS causes accumulation of the reduced form of electron carrier proteins and unused oxygen, resulting in uncoupling of mitochondria and conversion of oxygen into ROS. Since the generation of ROS was observed so rapidly, the primary target of dieldrin could be mitochondria, as also shown previously [31].

ROS is known to induce release of cytochrome c in neuronal cells. The release of cytochrome c was dose- and time-dependent, and occurred as early as 15 min following dieldrin exposure. The release of cytochrome c and other pro-apoptotic factors, such as apaf-1 from mitochondria, activates caspase-9 [47,48]. Caspase-9 serves as an initiator caspase, and it further proteolytically cleaves and activates effector caspases including caspase-3, -6, and -7 [49]. We observed a significant increase in caspase-3 activity following a 3 hr dieldrin exposure in a dose-dependent manner (100 and 300 μM dieldrin), indicating the level of dieldrin-induced cytochrome c release can promote the mitochondrial-mediated apoptotic cell process in dopaminergic cells.

Accumulating evidence strongly suggests the pro-apoptotic role of PKCδ during apoptosis in neuronal systems [43,44,50]. We have verified that the proteolytic activation of PKCδ was due to caspase-3 activation. The caspase-3 specific inhibitor Z-DEVD-FMK blocked the proteolytic cleavage of PKCδ by 70%, indicating the majority of PKCδ cleavage was due to caspase-3. Previously, we also showed that PKCδ plays an essential role in environmental chemical-induced apoptotic cell death in PC12 cells [32,37]. In these reports, PKCδ not only facilitates the downstream apoptotic process, including DNA fragmentation, but also modulates the upstream process, including caspase-3 activity, by an unknown mechanism. The regulatory role of PKCδ has also been documented elsewhere [44], but the exact regulatory function of PKCδ and mechanism by which it acts remain to be elucidated.

In the present paper, we focused on the execution of PKCδ. We demonstrated dieldrin-induced apoptosis using annexin-V-FITC. Further experiments characterized whether PKCδ played an important role in dieldrin-induced DNA fragmentation using catalytically inactive PKCδ mutant (PKCδ^K376R^)-expressed dopaminergic cells and measuring DNA fragmentation by ELISA. The kinase activity of mutant cells was documented previously by our laboratory [32]. Mutant cells were partially protected following dieldrin exposure, indicating that PKCδ is modulating DNA fragmentation somehow. Furthermore, pretreatment with a PKCδ specific inhibitor rottlerin also dose-dependently reduced nuclear condensation. These results are consistent with our previously published results related to dopaminergic toxins: MMT [51], MPP^+ ^[52], 6-OHDA [53] and manganese [54]. Recently, we demonstrated that PKCδ is highly expressed in the substantia nigra of mouse brain [55]. PKCδ negatively regulates the activity of tyrosine hydroxylase, a rate limiting enzyme for dopamine synthesis in dopaminergic neurons [55]. We have also shown that PKCδ inhibitor rottlerin protects against MPTP-induced behavioral, as well as neurochemical and biochemical deficits in animal models of Parkinson's disease [56]. Therefore, the proteolytic activation of PKCδ observed in this study may contribute to dopaminergic degeneration observed during dieldrin neurotoxicity.

## Conclusion

In conclusion, we demonstrate that dieldrin is a potent inducer of apoptosis in dopaminergic neuronal cells. Compared with our previous data from non-neuronal dopaminergic PC12 cells, neuron-derived cells seem to be more sensitive to dieldrin toxicity. Future investigation of chronic dieldrin exposure in animal models will help elucidate dieldrin neurotoxicity and the pathogenesis of neurodegenerative disorders such as Parkinson's disease.

## Competing interests

The authors declare that they have no competing interests.

## Authors' contributions

AGK, proposed hypotheses, designed study and oversaw the experiments. MK performed most of the experiments and currently a Post-Doctoral Fellow, Department of Neurobiology & Behavior, University of California, Irvine. YY, performed the immunocytochemistry experiments and currently a Post-Doctoral Fellow Department of Neurology, The Johns Hopkins University, Baltimore, MD. VA was responsible for DNA fragmentation and flow cytometry experiments. AK was responsible for data interpretation and assembled the manuscript. All the authors have read and approved the final manuscript.
